# Simulating Vibronic Spectra without Born–Oppenheimer Surfaces

**DOI:** 10.1021/acs.jpclett.1c00073

**Published:** 2021-03-22

**Authors:** Kevin Lively, Guillermo Albareda, Shunsuke A. Sato, Aaron Kelly, Angel Rubio

**Affiliations:** † Max Planck Institute for the Structure and Dynamics of Matter and Center for Free-Electron Laser Science, Luruper Chaussee 149, 22761 Hamburg, Germany; ‡ Institute of Theoretical and Computational Chemistry, University of Barcelona, Martí i Franquès 1-11, 08028 Barcelona, Spain; ¶ Nano-Bio Spectroscopy Group and ETSF, Universidad del País Vasco, 20018 San Sebastían, Spain; § Center for Computational Sciences, University of Tsukuba, Tsukuba 305-8577, Japan; ∥ Department of Chemistry, Dalhousie University, Halifax B3H 4R2, Canada; ⊥ Center for Computational Quantum Physics (CCQ), Flatiron Institute, 162 Fifth Avenue, New York, New York 10010, United States

## Abstract

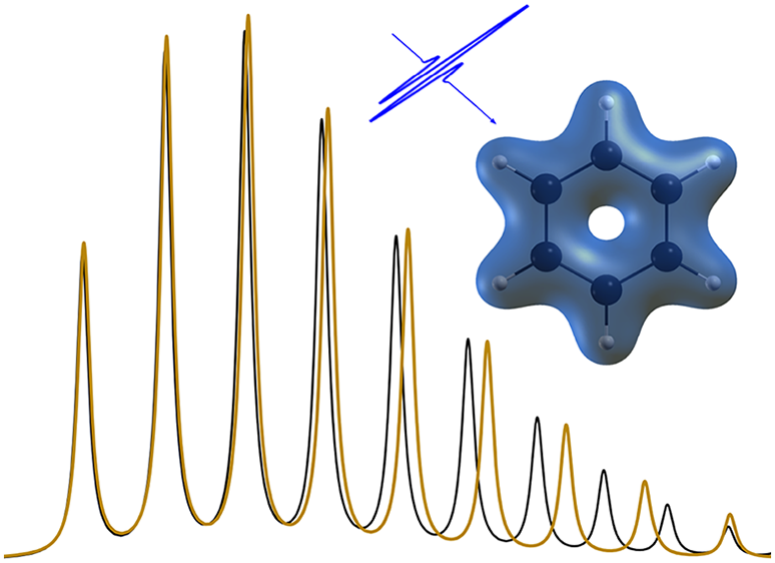

We show how linear vibronic spectra in molecular systems can be simulated efficiently using first-principles approaches without relying on the explicit use of multiple Born–Oppenheimer potential energy surfaces. We demonstrate and analyze the performance of mean-field and beyond-mean-field dynamics techniques for the H_2_ molecule in one dimension, in the later case capturing the vibronic structure quite accurately, including quantum Franck–Condon effects. In a practical application of this methodology we simulate the absorption spectrum of benzene in full dimensionality using time-dependent density functional theory at the multitrajectory Ehrenfest level, finding good qualitative agreement with experiment and significant spectral reweighting compared to commonly used single-trajectory Ehrenfest dynamics. These results form the foundation for nonlinear spectral calculations and show promise for future application in capturing phenomena associated with vibronic coupling in more complex molecular and potentially condensed phase systems.

Simulating vibronic effects from first-principles calculations is one of the central goals in theoretical spectroscopy that has implications in chemistry, physics, and materials science. The involvement of nuclear vibrational quantum states during electronic transitions plays a decisive role in determining the spectral features associated with these processes. This has been well-established by the utility of the Franck–Condon principle, for example, which represents an early paradigm for the role of nuclear quantum effects in electronically nonadiabatic processes. Describing this interplay between the electronic and vibrational degrees of freedom requires a quantum mechanical description that is both accurate and scalable to relatively large system sizes. One popular method to calculate vibronic spectra is to take a sum-over-states approach, where matrix elements of the transition operators between the states involved in generating the desired spectral signal are constructed. In this approach the states of interest can be represented using the Born–Oppenheimer (BO) basis; one must already have some *a priori* knowledge of the BO states that are involved, along with the associated potential energy surfaces and nonadiabatic couplings.

An alternative strategy to summing over states in the BO basis is to take a coordinate space perspective and construct the response function for the system of interest from direct time-propagation of the system in that picture.^1,2^ This invariably requires some level of approximation in the representation dynamics of the electronic and nuclear degrees of freedom, with different consequences for their coupling depending on the method chosen. The mixed quantum–classical Ehrenfest approach is a practical approximation to the fully quantum mechanical dynamics of the system, and despite its approximate *dynamics*, provides a formally exact representation of the quantum *equilibrium* structure of the correlated electronic and vibrational degrees of freedom via a multitrajectory Ehrenfest (MTEF) simulation through the use of the Wigner representation.^3−5^ In this case, the Wigner transform maps the vibrational quantum states onto phase space distributions of continuous position and momentum coordinates which can be sampled by an appropriate Monte Carlo procedure to capture the quantum equilibrium structure of the problem. The limitations of the Ehrenfest approach and other independent trajectory semiclassical methods are well-known,^6−10^ and while there have been many attempts to ameliorate these shortcomings, with some exceptions,^11,12^ most rely on the BO framework in their implementation.^13−17^ In this work we take a different approach to go beyond mean-field theory based on the recently introduced interacting conditional wave function (ICWF) formalism, which is able to capture correlated electronic and nuclear dynamics.^18−21^ We apply MTEF and ICWF dynamics to an exactly solvable one-dimensional H_2_ model and show that these methods are able to recover electron–nuclear correlations in linear vibronic spectra *without the need to calculate multiple BO surfaces*. In addition, we show that the MTEF method can be easily extended to *ab initio* nonadiabatic molecular dynamics simulations by calculating the vibronic spectra for benzene, where we find good agreement with experimental results.

The linear spectrum of a system is given by the Fourier transform of time correlation function (TCF) *C*_*AB*_(*t*) = ⟨[*Â*(*t*), *B̂*]⟩ of the transition dipole operator, μ̂, *C*_*μμ*_(*t*) = ⟨μ̂(*t*)μ̂(0)⟩^1,22^ (unless otherwise stated all expressions are in atomic units):
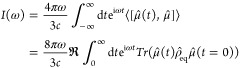
1where the trace occurs over nuclear and electronic degrees of freedom; ρ̂_eq_ is the equilibrium density matrix for the coupled system, and we evolve μ̂(*t*) in the Hilbert representation. Traditionally, vibronic spectra are explained by invoking the Franck–Condon approximation in the BO picture, where the electronic system is instantly excited, thus promoting the unperturbed ground-state nuclear system to a different electronic surface. If one has access to the electronic states involved in a particular spectral range then the contributions to the spectrum due to each electronic transition can be identified by resolving the transition dipole operator in the basis of the electronic states of interest, and the vibronic side peaks of that transition can be calculated by propagating the initial state’s nuclear subsystem under the effect of the nonequilibrium electronic occupation. When it is feasible to resolve the nuclear wave function dynamics, this can be one of the most accurate methods of calculating molecular vibronic spectra.^23,24^

Although resolving eq 1 in the BO framework is a powerful analysis tool, it is computationally impractical for systems with many nuclear degrees of freedom, particularly when one desires spectra over multiple surfaces. One can bypass this computational bottleneck by representing the system in a real space basis and using the “δ-kick” method,^25^ which captures electronic transitions to all dipole-transition allowed states (resolved on the grid) within a single calculation by utilizing the dipole response to a perturbative, but impulsive external field *Ĥ*_field_ = *E*(*t*)μ̂, with *E*(*t*) = *κδ*(*t*) and κ ≪ 1. Using first-order perturbation theory, the dipole response ⟨*Δμ*(*t*)⟩ = ⟨μ(*t*)⟩ – ⟨μ(0)⟩ can be written in powers of the field^1,2^
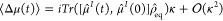
2where μ̂^*I*^(*t*) is evolved in the interaction representation. Hence, the linear response spectra may also be obtained via the relation
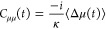
3provided the strength of the perturbing field, κ, is sufficiently small. This δ-kick approach requires only the initial state of the full system as input, followed by time propagation for a sufficient duration so as to obtain the desired energy resolution. Importantly, this technique can also serve as a foundation for calculating nonlinear optical response spectra.^26^

While the methods described above are formally equivalent, differences between the calculated spectra can arise when approximations are made. Here we briefly describe two methods for performing coupled electron nuclear dynamics simulations: the quantum–classical mean-field MTEF method and the ICWF formalism, which was designed to go beyond the mean-field limit.

A typical approach to Ehrenfest theory is to assume a separable electronic–nuclear wave function ansatz, take the classical limit of the nuclear portion, and initialize the nuclei at the equilibrium position with zero nuclear momentum.^27,28^ This single-trajectory Ehrenfest (STEF) method is often employed when a mixed quantum–classical method is needed to couple electronic and nuclear dynamics,^29^ in some cases providing a stark difference in electronic dynamics compared to fixed nuclei.^30,31^ Although attempts at capturing quantized vibrational effects in STEF with the δ-kick method have been made,^32^ they can contain unphysical spectral features (see the Supporting Information) which make them unsuitable for application to nonlinear spectra

An alternative route to Ehrenfest is also possible in the density matrix picture and proceeds via the quantum–classical Liouville equation.^33^ The major difference is that this representation results in a *multitrajectory Ehrenfest* picture of the dynamics, where the initial quantum statistics of the correlated system can, in principle, be captured exactly. Here, we outline the evolution equations, and we offer more details in the Supporting Information. The time evolution of the reduced electronic density is
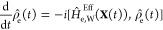
4where the subscript W refers to the partial Wigner transform over the nuclei; **X** = (**R**,**P**) is a collective variable for the nuclear position **R** and momentum **P**, and the effective electronic mean-field Hamiltonian is *Ĥ*_e,W_^Eff^(**X**(*t*)) = *Ĥ*_e_ + *Ĥ*_en,W_(**X**(*t*)), where *Ĥ*_e_ refers to the electronic portion of the Hamiltonian and *Ĥ*_en_ to the electron nuclear coupling. The nuclear dynamics is represented as an ensemble of *N* independent Wigner phase-space trajectories, ρ_n,W_(**X**,*t*) = 1/*N*∑_*i*_^*N*^δ(**X**_*i*_ – **X**_*i*_(*t*)), that evolve according to Hamilton’s equations of motion generated from the effective nuclear mean-field Hamiltonian
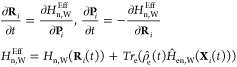
5

The average value of any observable, ⟨*O*(*t*)⟩, can then be written as
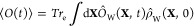
6which can be evaluated by sampling initial conditions from ρ̂_W_(**X**,0) and evolving the expectation value of the observable according to the above equations of motion. Using this dynamics method in conjunction with the BO basis representation to evaluate eqs 1 and 4–6 ultimately leads to the following equations of motion, with sums over BO states denoted by *a* (see the Supporting Information for details)
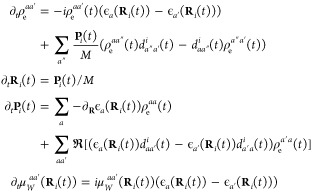
7where ϵ_*a*_(**R**) are the BO surfaces and *d*_*aa*′_ are the nonadiabatic coupling vectors (NACVs) between states *a* and *a*′.

In contrast to the previous expression, utilizing MTEF in the real space δ-kick approach requires initializing the electronic wave function as the BO eigenstate for each initially sampled nuclear geometry. The δ-kick is applied and the electronic wave function is propagated using the time-dependent Schrödinger equation equivalent to eq 4 alongside the nuclei according to eq 5. Calculating the spectrum via MTEF dynamics in the BO picture is from here on referred to as MTEF-BO, and calculating it via the δ−kick method is referred to as MTEF-kick.

Moving beyond semiclassical dynamics, the formally exact CWF method and its practical ICWF implementation are recently developed methods which have shown to be able to capture nonequilibrium correlated nuclear–nuclear and electron–nuclear phenomena beyond the mean-field limit.^18−21^ This approach is based on taking single-particle slices (the CWFs) of the time-dependent wave function of the full system; approximating the equations of motion for these CWFs by the Hermitian components of the sliced Hamiltonian; and finally, in the ICWF extension, utilizing these electron–nuclear CWFs as a basis of Hartree products in a wave function ansatz.

Here we describe an implementation of this approach utilizing the static and time-dependent variational principles for the expansion coefficients in a *static* CWF basis. The basis is chosen via sampling electronic and nuclear positions (**r**^α^, **R**^α^), α ∈ {1,···, *N*_c_}, where **r** and **R** are understood to be collective position variables, from initial guesses to the electronic and nuclear densities. These are used to construct the Hermitian limit of the CWF propagators^18^
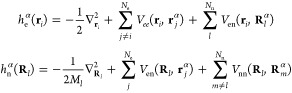
8for a system with *N*_e_ electrons and *N*_n_ nuclear degrees of freedom. Taking eigenstates of *h*_e_^α^(**r**_*i*_) and *h*_n_^α^(**R**_*l*_), denoted ϕ^α^(**r**_*i*_) and χ^α^(**R**_*l*_). respectively, as our (static) CWF basis we write the following single-index, multiconfigurational, mixed-species, wave function ansatz:
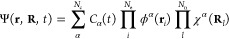
9where we have taken a Hartree product of electronic and nuclear CWFs for each degree of freedom. While the Hartree product over electronic degrees of freedom and a single expansion index has been sufficient for accuracy in applications of ICWF so far, this ansatz can in principle be trivially extended to a multi-index expansion and to have Fermionic antisymmetry via inclusion of Slater determinants. We then utilize the Dirac–Frenkel variational procedure^34−36^ to develop equations of motion for *C⃗*(*t*), which leads to the following standard evolution equation for the expansion coefficients of a nonorthogonal static basis:
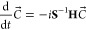
10where
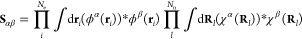

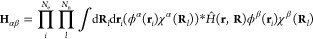
for the full molecular Hamiltonian *Ĥ*.

While the general form of this wave function ansatz is not unique, the mixed-species CWF basis treats the electronic and nuclear subsystems on an equal footing without relying on any adjustable parameters, using the Hermitian limit of the solutions to the conditionalized time-independent Schrödinger equations.

The ground-state wave function is obtained from this approach using imaginary time evolution,^37,38^ and the δ-kick spectra (ICWF-kick) is calculated by applying the perturbative field to the CWFs at time zero and recalculating the **S** and **H** matrices, equivalent to propagating in the interaction representation. In practice, **S** may be nearly singular, but its inverse can be approximated by the Moore–Penrose pseudoinverse.^39^ This “closed-loop” of initial state preparation and time-propagation ensures that our ICWF approach is a fully self-consistent method that increases in accuracy with increasing *N*_c_ and requires no BO state information.

To investigate the performance of the MTEF and ICWF approaches to vibronic spectral lineshapes we studied the vibronic transitions in an exactly solvable one-dimensional model system for molecular hydrogen.^40−42^ The total Hamiltonian can be written in the center of mass frame in atomic units as
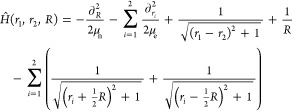
11where μ_n_ = *m*_p_/2 and μ_e_ = 2*m*_p_/(2*m*_p_ + 1) are the reduced nuclear and electronic masses; *R* is the internuclear separation, and *r*_*i*_ are the electronic positions. We take the proton mass to be *m*_p_ = 1836. The electronic and nuclear degrees of freedom were each resolved on grids for the numerically exact solution and ICWF-kick approaches, while the MTEF-kick electronic wave functions were time-evolved on the (*r*_1_, *r*_2_) grid, and the MTEF-BO information was calculated by solving the electronic subsystem across the nuclear grid; see Computational Methods for more details. A kick strength of κ = 10^–4^*a*_0_^–1^. was sufficient to generate the kick spectra within the linear response regime and, unless otherwise stated, a total propagation time of 10 000 au ≈ 242 fs was used to generate the spectra.

In Figure 1 we show mean-field spectra calculated both with (MTEF-BO) and without (MTEF-kick) the use of multiple BO surfaces for the absorption from *S*_0_ to *S*_2_ in comparison with the numerically exact results. We see that in the BO picture the MTEF method recovers the vibronic absorption peak placement quite accurately for the first five peaks, with a broadening occurring for the higher-energy peaks that leads to a loss of structure. This broadening of the spectral signal is due to the well-known fact that the MTEF dynamics does not preserve the correct quantum statistics and thus cannot fully capture the electron–nuclear correlation in the problem (see the Supporting Information for a detailed discussion of this issue). The prepeak features in Figure 1b are also unphysical artifacts of MTEF. The MTEF-BO spectra were converged to within graphical accuracy using *N* = 50 000 trajectories, although an ensemble size of approximately *N* = 500–1000 also yields reasonable results.

**Figure 1:**
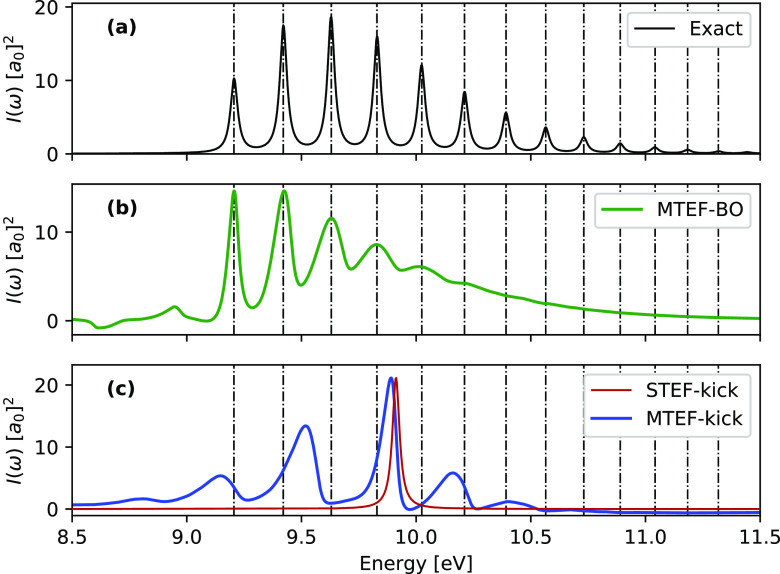
1D H_2_, *S*_2_ ← *S*_0_ spectra calculated via the MTEF-TCF, MTEF-kick, and STEF-kick approaches, with the exact peak placements overlaid as dashed vertical lines. Spectral cross sections are reported in square Bohr radii *a*_0_^2^. For clarity the STEF-kick spectrum has been multiplied by a factor of 0.175 to match the scale of the MTEF-kick results.

Focusing on the MTEF-kick results in Figure 1c, we see that this approach recovers vibronic side peak structures again without any BO surface information, albeit with inaccurate spacing, while STEF-kick captures only the vertical electronic transition from the minimum of the *S*_0_ surface. The average peak spacing in the MTEF-kick spectra is approximately 0.32 eV; this corresponds remarkably well with the natural frequency of the harmonic approximation to the ground-state surface expanded around the equilibrium geometry, which is also 0.32 eV in this case. This result is unsurprising as the electronic kick induces a very small population transfer to the upper surface proportional to the square of the kick strength, which results in the mean forces on the nuclei in MTEF-kick essentially corresponding to those of the initial state.

The influence of the initial state on the MTEF-kick spectra is further demonstrated by analyzing the emission spectra in Figure 2. The initial state here was chosen by hand as the lowest-lying nuclear state on the *S*_2_ surface. Once again we see that MTEF-BO recovers the peak placement quite well, while the MTEF-kick data has a less accurate vibronic spacing. Fitting the MTEF-kick peaks, we find an excellent correspondence between mean spacing of the five lowest-energy MTEF peaks and the excited surface natural frequency of 0.21 eV. Although the nuclear dynamics within MTEF-kick are primarily governed by the properties of the initial electronic state, the electron–nuclear coupling modulates the electronic linear response in a nontrivial manner, fundamentally changing the system response calculation compared to simply averaging the electronic transition properties over the equilibrium nuclear configuration, as is done in the nuclear ensemble approach^43^ (see the Supporting Information).

**Figure 2:**
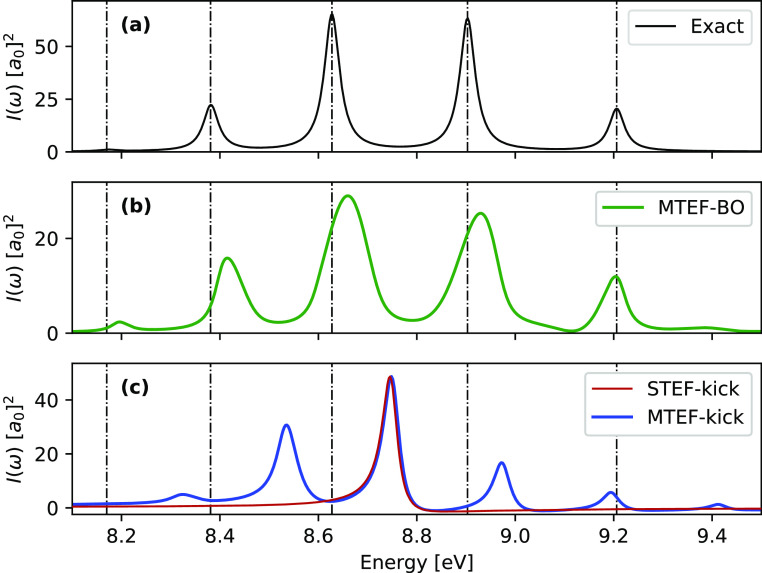
*S*_0_ ← *S*_2_ spectra compared between the MTEF-TCF, MTEF-kick, and STEF-kick approaches, with exact peak placement overlaid as dashed vertical lines. MTEF nuclear initial conditions are sampled from the lowest-lying vibrational state on *S*_2_. The sign of all spectra here is inverted for ease of comparison to other figures, and for legibility the STEF-kick spectrum was multiplied by a factor of 0.4 to match the MTEF-kick spectra maximum.

For ICWF-kick, we found that *N*_c_ = 4096 and mixing the three lowest-energy CWF eigenstates in roughly equal proportion was sufficient to obtain quite accurate results. In Figure 3 we demonstrate that the ICWF ansatz used in a variational context achieves a much more accurate vibronic spacing than the MTEF-kick approach, without the failing of peak broadening or unphysical spectral negativity apparent in the MTEF-BO results. The accuracy of these results underscores that the ICWF ansatz is a robust framework to capture the electronic and vibronic quantum dynamics, being accurate for not only the electron–nuclear correlation inherent to vibronic spectra but also the electronic subsystem itself, which in the MTEF results was solved exactly either on a grid or using explicit BO state information. The deviation from the exact results does grow with increasing energy, although this is ameliorated with increasing *N*_c_ and can in principle be eliminated at large enough values of *N*_c_ (see the Supporting Information).

**Figure 3:**
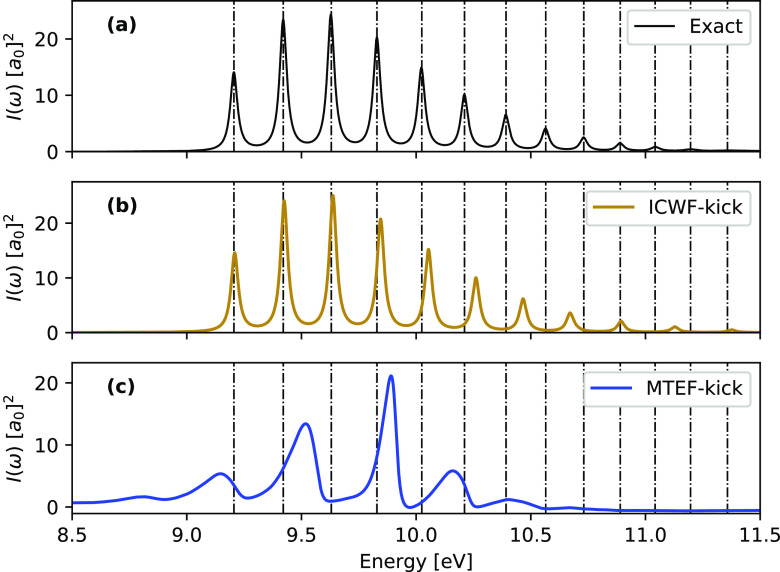
*S*_2_ ← *S*_0_ spectra of the ICWF-kick and MTEF-kick methods, with the exact peak placement overlaid as dashed lines.

Finally we demonstrate the application of MTEF-kick to real 3D molecular systems using the *ab initio* Octopus^44^ real-space time-dependent density functional theory (TDDFT)^45^ package to calculate the linear vibronic MTEF-kick spectra of benzene. The initial conditions for the nuclear subsystem were obtained by calculating the normal-mode frequencies and dynamical matrix of the molecule and sampling Wigner transforms of the ground-state wave functions in the harmonic approximation (see the Supporting Information for more details). The adiabatic-LDA functional was used, along with norm-conserving Troullier–Martins pseudopotentials, and the trajectories were evolved for  ≈ 132 fs with a time step of  ≈ 1 as. A kick strength of κ = 5 × 10^–3^ Å^–1^ was used to generate the kick spectra within the linear response regime in this case, and the graphical convergence of the MTEF results was found to be achieved with *N* = 500 trajectories.

In Figure 4, we compare the MTEF-TDDFT-kick results, its STEF-TDDFT-kick counterpart, and gas-phase experimental data.^46^ There is remarkably good agreement across the wide energy range available from experiment, before molecular dissociation pathways become available around 13.8 eV. Again, this full linear absorption spectrum is obtained without resorting to the calculation of individual transitions between states as would be required in a BO-state-based calculation. Principally, there is a significant spectral reweighting between STEF and MTEF below 17.5 eV, above which the electronic density of states is so high as to obscure the difference between the two methods. In the inset of Figure 4, in the 7 eV region corresponding to the energy range of the doubly degenerate, dipole-allowed , π* ← π transition,^31,47,48^ the STEF spectral weight is distributed across a much wider energy range in the MTEF signal, encompassing the experimental bands from 6 to 8 eV. The two STEF peaks at 8.5 and 8.95 eV are also spread across the 8–9 eV range. It is reasonable to expect that the broadening of the MTEF signal relative to the experimental signal is due to the effects discussed above that arise because of the mean-field treatment. In the Supporting Information we also compare these results to the broadening from the nuclear ensemble average calculation of the spectrum and find good agreement, given the high density of electronic states and many nuclear degrees of freedom in this system. By comparison with the standard STEF dynamics results used in large *ab initio* simulations, we see that utilizing multiple trajectories with equilibrium quantum nuclear statistics fundamentally changes the properties of the spectrum.

**Figure 4:**
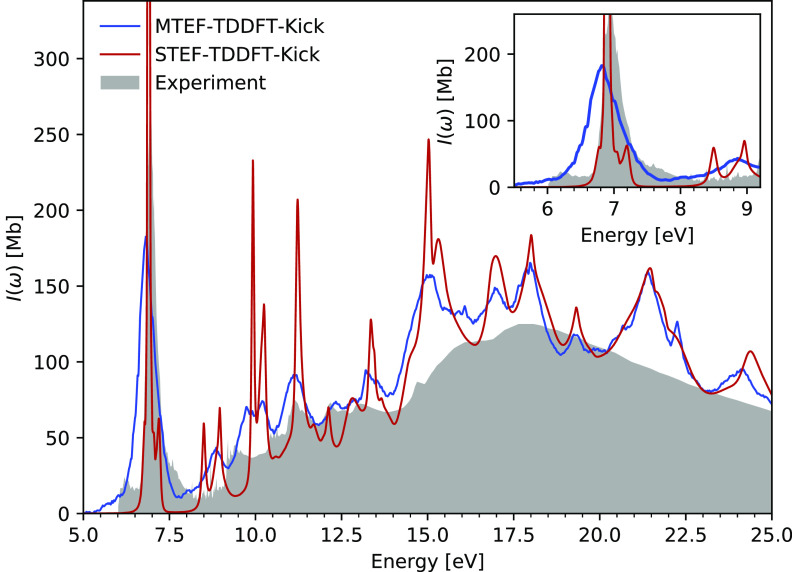
Experimental vibronic spectra for the lowest-lying optical transitions of benzene^46^ compared to the MTEF and STEF kick spectra calculated with TDDFT.

We have demonstrated that semiclassical MTEF simulations can capture vibronic structure with the correct spectral sign in the region of the transition. Moreover, we have shown how this can be achieved without using multiple BO surfaces via the δ-kick method and that the vibronic spacing calculated with the MTEF-kick approach matches the profile of the initial state. We have shown that utilizing a dynamics method that can accurately capture the correlated electron–nuclear dynamics, such as the ICWF method, in tandem with the δ-kick approach allows one to accurately recover the vibronic spectra. Finally, we demonstrated that MTEF-kick is easily applied to *ab initio* molecular systems by simulating the vibronic spectra of benzene and finding good agreement to experimental results.

These linear response results establish a solid basis for further investigations into nonlinear response of field driven molecular systems utilizing the practical and efficient MTEF and ICWF techniques along with *ab initio* electronic structure methods. Work in preparation by the present authors also explores the utility of ICWF with electron–electron and electron–nuclear correlated systems and explores the response of these systems under nonperturbative electric fields. Furthermore, we expect that MTEF-kick will improve in accuracy for periodic systems, as changes in the electronic configuration are often to likely produce smaller changes in the nuclear forces than in molecular hydrogen. This makes this method interesting to pursue in periodic systems in particular, where there is a dearth of theoretical frameworks for *ab initio*, nonpertubrative electron–nuclear coupling.^49^ Work in this direction is in progress, as is the implementation of the ICWF method within an *ab initio* framework for molecular and periodic systems.

## Computational Methods

In the 1D H_2_ model, the electronic coordinates are each resolved on a 65*a*_0_ wide interval with spacing of 0.6*a*_0_, while the nuclear grid extends to *R*_max_ = 6.3125*a*_0_ with 0.0625*a*_0_ spacing. Quadratic complex absorbing potentials were also added to the Hamiltonian to prevent reflection from the simulation box edge (see the Supporting Information). To generate the exact results we evolved the full wave function under the δ-kick on the three-dimensional electron–nuclear grid, while for MTEF-kick, the electronic subsystem’s Schrödinger equation, dependent on **R**_*i*_(*t*), was solved exactly on the two-dimensional electronic grid for each trajectory. All wave functions were time-propagated using a fourth-order Runge–Kutta integration scheme with a time-step size of *Δt* = 0.05 au. For the MTEF trajectories, the nuclear degree of freedom was propagated via a velocity Verlet type scheme with the same time-step size.^50^ An exponential damping mask function exp(−*γt*) was applied to all time-dependent signals in the Fourier transform, and the damping factor was set to damp the signal to 0.1% of its strength at the final time.

For the 1D H_2_ MTEF-BO results, the potential energy surfaces ϵ_*a*_(*R*) and μ_W_^*aa*′^ (*R*) were calculated on a nuclear grid with *ΔR* = 0.02*a*_0_ up to *R*_max_ = 8*a*_0_, fit to a cubic spline function, and interpolated every 0.01*ΔR*. The NACV between *S*_0_ and *S*_2_ in this model is numerically zero. These quantities were resolved for the first allowed dipole transition, between the ground state (*S*_0_) and the second excited state (*S*_2_), and the results were found to be well converged within about 5 × 10^4^ trajectories.

For the MTEF-TDDFT-kick simulations we used a real space grid formed from overlapping spheres of radius 8 Å centered on the initial positions of the nuclei, with an isotropic grid spacing of 0.16 Å, which was found to be sufficient to converge the energies of the lowest-lying absorption lines. The reported results were calculated on a hyperthreaded 16 CPU core Xeon E5-2698 v3 requiring approximately 880 core hours per trajectory. Being composed of independent trajectories the cost of the MTEF method over the STEF simulation scales linearly with the number of trajectories, requiring approximately 440 000 core hours for graphical convergence at 500 trajectories.

## Supporting Information Available

Details on MTEF, STEF, and ICWF as referenced in the main text (PDF)
