# Supplementary material for: Simulating Vibronic Spectra without Born–Oppenheimer Surfaces
Source: J Phys Chem Lett. 2021 Mar 22;12(12):3074–81. doi: 10.1021/acs.jpclett.1c00073 (PMC8020382; doi:10.1021/acs.jpclett.1c00073)
Supplement: Supplementary file 1 — jz1c00073_si_001.pdf [file jz1c00073_si_001.pdf]

Simulating Vibronic Spectra without Born-Oppenheimer Surfaces: Supplemental Information

Kevin Lively,[†] Guillermo Albareda,[†,‡,¶] Shunsuke A. Sato,[†,§] Aaron Kelly,[*,†,‖] and Angel Rubio[*,†,¶,⊥]

†*Max Planck Institute for the Structure and Dynamics of Matter and Center for Free-Electron Laser Science, Luruper Chaussee 149, 22761 Hamburg, Germany*

‡*Institute of Theoretical and Computational Chemistry, University of Barcelona, Martí i Franquès 1-11, 08028 Barcelona, Spain*

¶*Nano-Bio Spectroscopy Group and ETSF, Universidad del País Vasco, 20018 San Sebastían, Spain*

§*Center for Computational Sciences, University of Tsukuba, Tsukuba 305-8577, Japan*

‖*Department of Chemistry, Dalhousie University, Halifax B3H 4R2, Canada*

⊥*Center for Computational Quantum Physics (CCQ), Flatiron Institute, 162 Fifth avenue, New York NY 10010, USA*

E-mail: aaron.kelly@mpsd.mpg.de; angel.rubio@mpsd.mpg.de

MTEF Equations of Motion

Starting from a density matrix representation of the full system, $\hat{\rho}$, we Wigner transform over the nuclear subsystem, producing a unique mapping onto a nuclear position $\mathbf{R}$ and momentum $\mathbf{P}$ phase space $\mathbf{X} = (\mathbf{R}, \mathbf{P})$, where $\mathbf{R}$ and $\mathbf{P}$ are collective variables $\mathbf{R} = (\mathbf{R}_1, \ldots, \mathbf{R}_{N_n})$, $\mathbf{P} = (\mathbf{P}_1, \ldots, \mathbf{P}_{N_n})$, with $\mathbf{R}_i, \mathbf{P}_i \in \mathbb{R}^d$. The partial wigner transform is defined for any operator as

$$\hat{\rho}_W(\mathbf{R}, \mathbf{P}) = \frac{1}{(2\pi)^{dN_n}} \int d\mathbf{X} e^{i\mathbf{P}\cdot\mathbf{X}} \langle \mathbf{R} - \frac{\mathbf{X}}{2} | \hat{\rho} | \mathbf{R} + \frac{\mathbf{X}}{2} \rangle , \tag{1}$$

leaving a Hilbert space operator character over the electronic degrees of freedom, dependent on the continuous nuclear phase space parameters. In general, developing equations of motion for $\hat{\rho}_W(\mathbf{R}, \mathbf{P})$, (or any operator), requires taking the partial Wigner transformation of the Liouville von-Neumann equation of motion for ρ:

$$\begin{aligned}
\frac{\partial \hat{\rho}_W}{\partial t} &= -i\Big((\hat{H}\hat{\rho})_W - (\hat{\rho}\hat{H})_W \Big) \\
(\hat{H}\hat{\rho})_W &= \hat{H}_W \exp\left(\frac{1}{2i}\Lambda\right) \hat{\rho}_W \\
\Lambda &= \overleftarrow{\nabla}_{\mathbf{P}} \cdot \overrightarrow{\nabla}_{\mathbf{R}} - \overleftarrow{\nabla}_{\mathbf{R}} \cdot \overrightarrow{\nabla}_{\mathbf{P}} \\
g \exp\Big(\kappa\Lambda\Big) f &= \sum_{s=0}^{\infty} \frac{\kappa^s}{s!} \sum_{t=0}^{s} (-1)^t \binom{s}{t} \left[\partial_{\mathbf{R}}^{s-t} \partial_{\mathbf{P}}^{t}\, f\right] \left[\partial_{\mathbf{R}}^{t} \partial_{\mathbf{P}}^{s-t}\, g\right] .
\end{aligned} \tag{2}$$

Where the final line defines the "Moyal product" also known as the "star product".[1] By expressing the Poisson braket operator Λ, in terms of the ratio of masses between the nuclei and the electrons $\Lambda = (m/M)^{\frac{1}{2}}\Lambda'$, and truncating the Moyal product of $e^{(m/M)^{\frac{1}{2}}\Lambda'}$ at first order, one can arrive at the Quantum-Classical Liouville Equation (QCLE):[2]

$$i\frac{\partial}{\partial t}\hat{\rho}_W(\mathbf{R}, \mathbf{P}) = -i[\hat{H}_W, \hat{\rho}_W] + \frac{1}{2}\Big(\{\hat{H}_W, \hat{\rho}_W\} - \{\hat{\rho}_W, \hat{H}_W\}\Big), \tag{3}$$

where $\{A(\mathbf{R}, \mathbf{P}), B(\mathbf{R}, \mathbf{P})\}$ refers to the normal Poisson bracket.

To derive MTEF equations of motion from the QCLE, one takes the mean field approximation by assuming that the full system can be written as a sum of correlated and uncorrelated parts,

$$\hat{\rho}_W(\mathbf{X},t) = \hat{\rho}_e(t)\rho_{n,W}(\mathbf{X},t) + \hat{\rho}_{corr,W}(\mathbf{X},t), \tag{4}$$

and then neglecting the contribution of the correlated part in the *dynamics.* Note that while the ensuing dynamics do not explicitly treat the effect of subsystem correlation, the initial state generally *is* correlated, and therefore is implicitly included in the dynamics.

Under this approximation, the electronic density matrix is

$$\hat{\rho}_e(t) = Tr_n\Big(\hat{\rho}(t)\Big) = \int d\mathbf{X}\hat{\rho}_W(\mathbf{X},t), \tag{5}$$

and the nuclear (quasi) probability phase space distribution is $\rho_n(\mathbf{X},t) = Tr_e\left(\hat{\rho}_W(\mathbf{X},t)\right)$.

In the equations of motion resulting from inserting this approximation into the QCLE, the evolution of the reduced Wigner density of the nuclear subsystem can be exactly represented, via the method of characteristics, by a sufficiently large ensemble of multiple independent trajectories, $\rho_{n,W}(\mathbf{X},t) = \frac{1}{N}\sum_i^N \delta(\mathbf{X}_i - \mathbf{X}(t))$. Each trajectory evolves according to Hamilton's equations of motion generated from the mean-field effective Hamiltonian,

$$\begin{aligned} \frac{\partial \mathbf{R}_i}{\partial t} &= \frac{\partial H^{Eff}_{n,W}}{\partial \mathbf{P}_i}, \quad \frac{\partial \mathbf{P}_i}{\partial t} = -\frac{\partial H^{Eff}_{n,W}}{\partial \mathbf{R}_i} \\ H^{Eff}_{n,W} &= H_{n,W}(\mathbf{X}_i(t)) + Tr_e\Big(\hat{H}_{en,W}(\mathbf{X}_i(t))\hat{\rho}^i_e(t)\Big). \end{aligned} \tag{6}$$

Where $H_{n,W}$ and $H_{en,W}$ refer to the partially Wigner transformed nuclear and electron-nuclear coupling operators, respectively. The electronic density associated with each trajectory , $\rho^i_e(t)$, evolves according to the following commutator:

$$\frac{d}{dt}\hat{\rho}^i_e(t) = -i\Big[\hat{H}_e + \hat{H}_{en,W}(\mathbf{X}_i(t)),\ \hat{\rho}^i_e(t)\Big]. \tag{7}$$

The exact expression for the average value of any observable, $\langle O(t)\rangle$, can be written as

$$\begin{aligned}\langle O(t)\rangle &= Tr_e\int d\mathbf{X}\hat{O}_W(\mathbf{X},t)\hat{\rho}_W(\mathbf{X},0) = Tr_e\int d\mathbf{X}\hat{O}_W(\mathbf{X})\hat{\rho}_W(\mathbf{X},t)\\ &= \frac{1}{N}\sum_i^N Tr_e\Big(\ \hat{O}_W(\mathbf{X}_i(t))\hat{\rho}_e^i(t)\ \Big)\end{aligned} \tag{8}$$

The mean field limit of this expression simple corresponds to evaluating the integral by sampling initial conditions for an ensemble of independent trajectories from $\hat{\rho}_W(\mathbf{X},0)$, and then generating the time evolution for each trajectory by approximating $\hat{O}_W(\mathbf{X},t)$ by it's mean-field counterpart.

Following the sampling of an initial nuclear condition, $\mathbf{X}_i$, from the Wigner distribution associated to the nuclear subsystem wave function, the electronic system is initialised as:

$$(\hat{H}_e + \hat{H}_{en,W}(\mathbf{R}_i))\phi_a(r) = \epsilon_a(\mathbf{R}_i)\phi_a(r), \tag{9}$$

i.e. implicitly as the BO electronic state at $\mathbf{R}_i$. Under this scheme, the electronic subsystem's initial conditions are implicitly correlated with the nuclear subsystem's quantum statistics.

In cases where the nuclear initial state is impractical to calculate exactly one may utilise the normal modes of the molecular system, or phonon coordinates of a periodic system, to treat the full nuclear wavefunction as a Hartree product of N uncoupled harmonic oscillators, where N is the number of non-rotational and non-translational nuclear degrees of freedom:

$$\begin{aligned}\chi_n(\mathbf{R}) &\approx \chi_1(Q_1)\otimes\ldots\otimes\chi_N(Q_N)\\ \chi_i(Q_i) &= \sum_l c_l^{(i)}\chi_i^l(Q_i).\end{aligned} \tag{10}$$

With $c_l^{(i)}$ referring to the occupation of the lth excited state of normal mode i with wavefunction, χ_i^l, and $Q_i(\mathbf{R})$ the normal mode coordinate. Formally, this is exactly equivalent to taking a second order Taylor expansion approximation of the BO surface about the equilib-

rium nuclear position R^0:

$$H_{nuc}(\mathbf{R},\mathbf{P}) = \sum_l \frac{1}{2M_l}\mathbf{P}_l^2 + \sum_{lm}\frac{1}{2}(\mathbf{R}_l - \mathbf{R}_l^0)\frac{\partial^2 V_{BO}}{\partial \mathbf{R}_l \partial \mathbf{R}_m}\bigg|_{\mathbf{R}^0}(\mathbf{R}_m - \mathbf{R}_m^0). \tag{11}$$

Defining the dynamical matrix, $\mathcal{H}_{lm} = \frac{1}{\sqrt{M_l}}\frac{\partial^2 V}{\partial \mathbf{R}_l \partial \mathbf{R}_m}\frac{1}{\sqrt{M_m}}$, and it's diagonalizing unitary transform, $D^T\mathcal{H}D = \Omega$, $D^T D = \mathbf{1}$, where $\Omega_{ij} = \omega_i^2\delta_{ij}$, we construct the normal coordinate transform for all non-rotational, non-translational (imaginary) ω_i^2, (here we include $\hbar$ for clarity):

$$\begin{gathered}\sqrt{M_l}(\mathbf{R}_l - \mathbf{R}_l^0) = \sum_i D_{li} q_i\ , \quad \frac{\mathbf{P}_l}{\sqrt{M_l}} = \sum_i D_{li} s_i \\ s_i = \sqrt{\hbar\omega_i} S_i\ , \quad q_i = \sqrt{\frac{\hbar}{\omega_i}} Q_i,\end{gathered} \tag{12}$$

such that we obtain the nuclear Hamiltonian in dimensionless normal mode coodinates:

$$H(Q,S) = \sum_i \frac{\hbar\omega_i}{2}(S_i^2 + Q_i^2). \tag{13}$$

Of course, the simple harmonic wave function solutions to the above Hamiltonian have well known analytical expressions and are trivially Wigner transformed, the ground state harmonic oscillator wavefunction's Wigner function for instance is:[3]

$$W_0(Q,S) = \frac{1}{\pi}\exp\left(-S^2 - Q^2\right). \tag{14}$$

We can therefore sample these transforms for (Q,S) and then use eq. (12) to back transform to from normal mode coordinates to cartesian coordinates.

MTEF-BO Equations of Motion in the Born Oppenheimer Basis

In deriving the MTEF equations of motion in the BO basis, we start by writing the molecular hamiltonian in terms of position and momentum space operators for the electrons (light particles), $\hat{r}, \hat{p}$ and nuclei (heavy particles) $\hat{R}, \hat{P}$. These are again understood to be collective variables.

$$\begin{aligned} \hat{H}(\hat{r},\hat{p},\hat{R},\hat{P}) &= \frac{1}{2M}\hat{P}^2 + \hat{h}_e(\hat{r},\hat{p},\hat{R}) \\ \hat{h}(\hat{r},\hat{p},\hat{R}) &= \frac{1}{2}\hat{p}^2 + \hat{V}(\hat{r},\hat{R}) \\ \hat{V}(\hat{r},\hat{R}) &= \hat{V}_{ee}(\hat{r}) + \hat{V}_{en}(\hat{r},\hat{R}) + \hat{V}_{nn}(\hat{R}). \end{aligned} \tag{15}$$

We then utilise a position representation in the nuclear dof by expanding in the space of nuclear position states $1_{\mathbf{R}} = \int d\mathbf{R} \left|\mathbf{R}\right\rangle \left\langle\mathbf{R}\right|$, leading to

$$\hat{H}(\mathbf{R}) = -\frac{1}{2M}\nabla^2_{\mathbf{R}} + \hat{h}_e(\hat{r},\hat{p},\mathbf{R}) \tag{16}$$

For a transition between two electronic states g and e, we can expand in the adiabatic basis $\left|\phi_a(\mathbf{R})\right\rangle, (a = g, e)$ which are dependent on the nuclear positions $\mathbf{R}$ defined by,

$$\hat{h}_e(\mathbf{R}) \left|\phi_a(\mathbf{R})\right\rangle = \epsilon_a(\mathbf{R}) \left|\phi_a(\mathbf{R})\right\rangle. \tag{17}$$

Taking the partial Wigner transform of eq. (16) leads to

$$\hat{H}_W(\mathbf{R},\mathbf{P}) = \frac{1}{2M}\mathbf{P}^2 + \hat{h}_{e,W}(\hat{r},\hat{p},\mathbf{R}) \tag{18}$$

where $\hat{h}_{e,W}(\mathbf{R})$ is the normal electronic hamiltonian operator, now dependent on R in the Wigner nuclear phase space. Starting with the separability approximation for the density

operator, and neglecting correlations, we have $\hat{\rho}_W = \hat{\rho}_e \rho_n(\mathbf{R}, \mathbf{P})$, with

$$\partial_t \hat{\rho}_e = -i \left[Tr_{\mathbf{X}} \langle \hat{h}_{e,W}(\mathbf{R}) \rangle , \hat{\rho}_e \right] \tag{19}$$

where $Tr_{\mathbf{X}} \langle \ldots \rangle = \int \ldots d\mathbf{R} d\mathbf{P}$, and $\mathbf{P}$ scalar terms are cancelled by the commutator. We are of course interested in evaluating the dipole-dipole correlation function:

$$\begin{aligned} C_{\mu\mu}(t) &= \int d\mathbf{R} d\mathbf{P} Tr_e \Big\{ \hat{\mu}_W \hat{\sigma}(t) \Big\} \\ &= \int d\mathbf{R} d\mathbf{P} Tr_e \Big\{ \hat{\mu}_W(t) \hat{\sigma}(0) \Big\}, \end{aligned} \tag{20}$$

where $\hat{\sigma} = [\hat{\mu}_W, \hat{\rho}_W]$, and we resolve the dipole operator as

$$\begin{aligned} \hat{\mu}_W(\mathbf{R}, t=0) &= -\hat{r} + Z_{\mathbf{R}} \mathbf{R} \\ &= \sum_{aa'} |\phi_a\rangle \langle \phi_a | (-\hat{r}) | \phi_{a'} \rangle \langle \phi_{a'} | + \delta_{aa'} Z_{\mathbf{R}} \mathbf{R} |\phi_a\rangle \langle \phi_{a'}| \\ &= \begin{pmatrix} \mathbf{R} & \mu^{ge}(\mathbf{R}) \\ \mu^{eg}(\mathbf{R}) & \mathbf{R} \end{pmatrix} \end{aligned} \tag{21}$$

Where $Z_{\mathbf{R}}$ refers to the ionic charge of each nuclei. In practice we can neglect the intra-state $\mathbf{R}$ term as we are focused entirely on the *transition* dipole moment.

Taking the initial state as the ground state, ($|\Psi\rangle = |\chi_g^0 \phi_g\rangle$)

$$\hat{\rho}_W(\mathbf{R}, \mathbf{P}, 0) = \rho_g^n(\mathbf{R}, \mathbf{P}) \begin{pmatrix} 1 & 0 \\ 0 & 0 \end{pmatrix}, \tag{22}$$

leads to

$$\begin{aligned}\hat{\sigma(0)} &= [\hat{\mu}_W, \hat{\rho}_W(\mathbf{R}, \mathbf{P}, 0)] \\ &= \rho_g^n(\mathbf{R}, \mathbf{P}) \begin{pmatrix} 0 & -\mu^{ge}(\mathbf{R}) \\ \mu^{eg}(\mathbf{R}) & 0 \end{pmatrix}.\end{aligned} \tag{23}$$

And therefore the correlation function becomes

$$\begin{aligned}C_{\mu\mu}(t) &= \int d\mathbf{R}d\mathbf{P}\left(\mu_W^{ge}(\mathbf{R}, t)\sigma^{eg}(0) + \mu_W^{eg}(\mathbf{R}, t)\sigma^{ge}(0)\right) \\ &= \int d\mathbf{R}d\mathbf{P}\big(\mu_W^{ge}(\mathbf{R}, t)\mu_W^{eg}(\mathbf{R}, 0) \\ &- \mu_W^{eg}(\mathbf{R}, t)\mu_W^{ge}(\mathbf{R}, 0)\big)\rho_g^n(\mathbf{R}, \mathbf{P}).\end{aligned} \tag{24}$$

We can construct an identical quantity from a different initial condition as a superposition state ($|\Psi\rangle = \frac{1}{\sqrt{2}}|\chi_g\rangle(|\phi_g\rangle + i|\phi_e\rangle)$) giving,

$$\hat{\tilde{\rho}}_W(\mathbf{R}, P, 0) = \rho_g^n(\mathbf{R}, \mathbf{P})\frac{1}{2}\begin{pmatrix} 1 & -i \\ i & 1 \end{pmatrix} \tag{25}$$

For this different initial condition we propagate

$$\begin{aligned}\tilde{C}_{\mu\mu}(t) &= \frac{i}{2}\int dRdP\Big(\mu_W^{ge}(\mathbf{R}, t)\mu_W^{eg}(\mathbf{R}, 0) - \mu_W^{eg}(\mathbf{R}, t)\mu_W^{ge}(\mathbf{R}, 0)\Big)\rho_g^n(\mathbf{R}, \mathbf{P}) \\ &= \frac{i}{2}C_{\mu\mu}(t)\end{aligned} \tag{26}$$

With this different initial condition, we take the MTEF form of the nuclear density arising from the Monte Carlo integration described above,

$$\rho_n(\mathbf{R}, \mathbf{P}) = \frac{1}{N}\sum_i \delta(\mathbf{R} - \mathbf{R}_i(t))\delta(\mathbf{P} - \mathbf{P}_i(t)). \tag{27}$$

The subsequent equations of motion for the system are for the electronic density, needed for

the nuclear trajectories are:

$$
\begin{aligned}
\partial_t \tilde{\rho}_e^{aa'} &= -i\tilde{\rho}_e^{aa'}(t)(\epsilon_a(\mathbf{R}_i(t)) - \epsilon_{a'}(\mathbf{R}_i(t))) \\
&\quad + \sum_{a''} \frac{\mathbf{P}_i(t)}{M} \left(\tilde{\rho}_e^{aa''}(t) d^i_{a''a'}(t) - d^i_{aa''}(t)\tilde{\rho}_e^{a''a'}(t) \right) \\
\partial_t \mathbf{R}_i(t) &= \mathbf{P}_i(t)/M \\
\partial_t \mathbf{P}_i(t) &= \frac{1}{2}\sum_{aa'} \left(F_W^{aa'}(t)\tilde{\rho}_e^{a'a}(t) + \tilde{\rho}_e^{aa'}(t) F_W^{a'a}(t) \right) \\
&= \sum_{aa'} \Re\left[F_W^{aa'}(t)\tilde{\rho}_e^{a'a}(t) \right] \\
&= \sum_{a} -\partial_R \epsilon_a(\mathbf{R}_i(t))\tilde{\rho}_e^{aa}(t) \\
&\quad + \sum_{aa'} \Re\left[\left(\epsilon_a(\mathbf{R}_i(t)) d^i_{aa'}(t) - \epsilon_{a'}(\mathbf{R}_i(t)) d^i_{a'a}(t) \right) \tilde{\rho}_e^{a'a}(t) \right]
\end{aligned}
\tag{28}
$$

Where in the last two equations we have used the identity $d^i_{aa'}(t) = \langle \phi_a | \partial_{\mathbf{R}_i} \phi_{a'} \rangle |_{\mathbf{R}_i(t)} = -(d^i_{a'a}(t))^*$, to manipulate $F_W^{aa'}(t) = -\langle \phi_a(\mathbf{R}) | \partial_R \hat{H}_W | \phi_{a'}(\mathbf{R}) \rangle |_{\mathbf{R}_i(t)}$. Note that for transitions like the S_0/S_2 transition 1D H_2 focused on in the body of this paper, the non-adiabatic coupling vector (NACV) $d_{aa'} = 0$, means that the mean field force acting on the nuclei is at all times a $\frac{1}{2}$ superposition of the S_0 and S_2 surfaces.

These are propagated alongside the dipole matrix element equations of motion, needed for the correlation function:

$$
\partial_t \mu_W^{aa'}(\mathbf{R}_i(t)) = i\mu_W^{aa'}(\mathbf{R}_i(t))(\epsilon_a(\mathbf{R}_i(t)) - \epsilon_{a'}(\mathbf{R}_i(t))). \tag{29}
$$

STEF Spectral Negativity

As mentioned in the main text, previous work by Goings et. al[4] employed STEF-kick dynamics simulations to calculate spectra in fully ab-initio 3D H_2 by initialzing the nuclear geometry in non-equilibrium 'compressed' geometries. Geometries were selected corresponding to expected vibrational energies from Boltzmann distributions at arbitrary temperatures

and the $\delta-$Kick method was used to excite the electronic subsystem. Furthermore, only the *magnitude* of the spectral response was depicted, which does not show the spectral negativity resulting from initialising the mean field simulations in a non-equilibrium state. Here we utilise the canonical initial conditions of the STEF-BO picture for the 1D H_2 model. The electronic occupation is equal for each of the two surfaces ivolved in the transition, and the nuclear initial condition corresponds to the equillibrium geometry of the initial surface. In

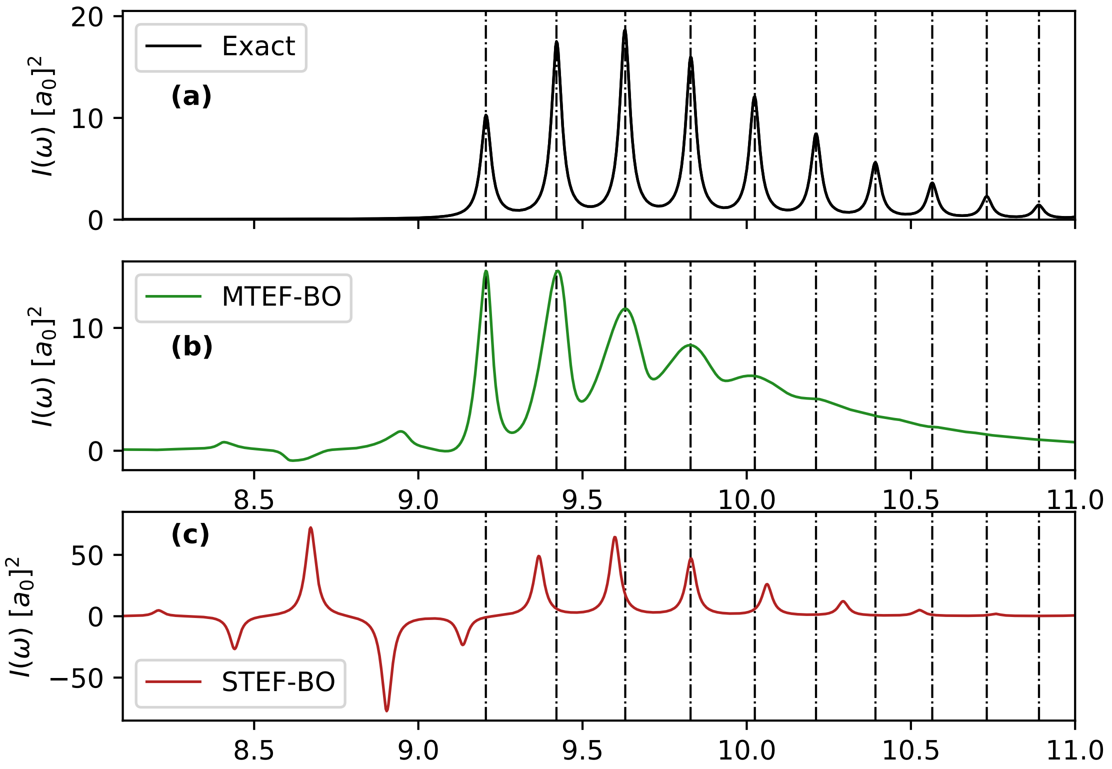

Figure S1: 1D H_2 $S_0 \leftarrow S_2$ absorption spectra, comparing exact, MTEF-BO and STEF-BO.

Fig. S1c we see the results of STEF-BO for the $S_2 \leftarrow S_0$ region of the spectrum, showing that this only captures positive spectral intensities in the vicinity of the exact results, with accurate peak placement only at the MTEF level. Furthermore the contributions to the unphysical pre-peak features of individual trajectories become apparent in the low energy tail. For completeness we also feature the $S_0 \leftarrow S_2$ results in Fig. S2, which demonstrate the same features of correct spectral sign only in the region of the exact results and alternating sign elsewhere.

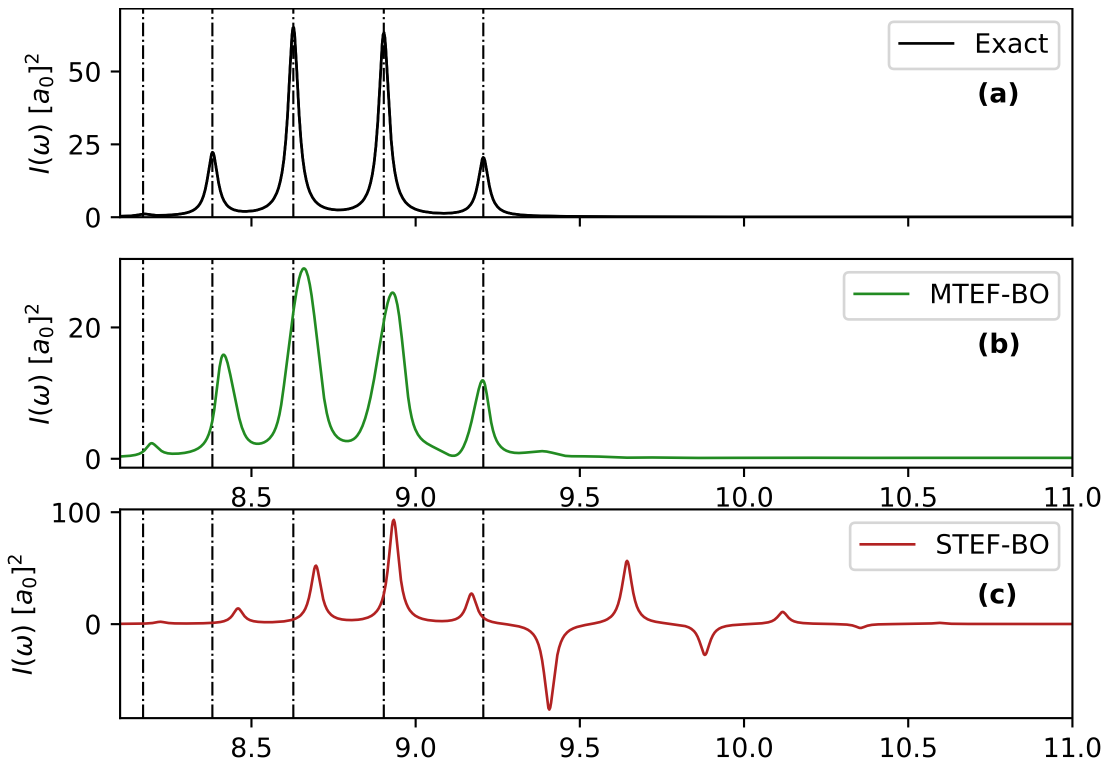

Figure S2: 1D H_2 $S_2 \leftarrow S_0$ absorption spectra, comparing exact, MTEF-BO and STEF-BO.

Application to Displaced Harmonic Oscillator Model

In order to investigate the limitations of MTEF, we can utilise a model which captures the essential physics of the S_0/S_2 1D H_2 transition which was focused on in the first portion of the main text. Recall that for this transition, the NACV's between the two electronic adiabatic states are zero, that is $\langle \phi_a(R) | \partial_R \phi_{a'}(R) \rangle = 0 \ \forall \ a, a'$ in the BO basis, with a, a' restricted to S_0/S_2 This means that matrix elements for the partially Wigner transformed molecular hamiltonian can be written as

$$\hat{H}_W(R,P) = \frac{P^2}{2M}1 + \begin{pmatrix} \epsilon_g(R) & 0 \\ 0 & \epsilon_e(R) \end{pmatrix}. \tag{30}$$

As described in detail in the first section of this SI, MTEF is rooted in a mean field approximation to the QCLE, which is itself the first order expansion of the partially Wigner

transformed Liouville von-Neumann equation. Taking eq. (2) to second order provides,

$$\begin{aligned}\frac{\partial \hat{\rho}_W}{\partial t} &= -i\left[\hat{H}_W, \hat{\rho}_W\right] \\ &+ \frac{1}{2}\left(\{\hat{H}_W, \hat{\rho}_W\} - \{\hat{\rho}_W, \hat{H}_W\}\right) \\ &- \frac{i}{8}\left(\left[\partial_P^2 \hat{H}_W, \partial_{\mathbf{R}}^2 \hat{\rho}_W\right] + \left[\partial_{\mathbf{R}}^2 \hat{H}_W, \partial_{\mathbf{P}}^2 \hat{\rho}_W\right]\right)\end{aligned} \tag{31}$$

Which in our model Hamiltonian eq. (30) becomes,

$$\begin{aligned}\frac{\partial \rho_W^{aa'}}{\partial t} &= -i(\epsilon_a(R) - \epsilon_{a'}(R))\rho_W^{aa'} \\ &+ \left[\frac{1}{2}\left(\partial_R \epsilon_a(R) + \partial_R \epsilon_{a'}(R)\right)\partial_P - \frac{P}{M}\partial_R\right]\rho_W^{aa'} \\ &- \frac{i}{8}\left(\partial_R^2 \epsilon_a(R) - \partial_R^2 \epsilon_{a'}(R)\right)\partial_P^2 \rho_W^{aa'} + \mathcal{O}\big((m/M)^{\frac{3}{2}}\big)\end{aligned} \tag{32}$$

Such that the error in time propagation resultant from taking only the first order expansion, compared to the second, is proportional to the difference in energy surface curvature.

If we take the analytically solvable Displaced Harmonic Oscillator (DHO) model[5,6] by using surfaces $\epsilon_a(R) = \frac{1}{2}\omega_a^2(R - D_a)^2 + E_a$, we see that for identical surfaces $\omega_e = \omega_g$ that the 2nd order and higher terms in the Wigner transformed Liouville von-Neumann equation are zero, rendering the QCLE exact for this case.

To demonstrate the effect of varying surface curvature, we took parameters similar to harmonic surface fits to the BO surfaces in 1D H_2, and for simplicity, took the FC approximation alongside setting $\mu^{aa'}(R) = (1 - \delta_{aa'})$a.u.. We solve the exact and MTEF-TCF spectra for the DHO with different values of ω_e relative to ω_g by propagating for $T_f = 2 \cdot 10^4$a.u.. In Fig. (S3) we see iin the left column that for identical upper and lower surfaces, mean field theory is of course exact, and for varying surfaces, MTEF displays a peak broadening and prepeak features. The origin of this broadening is from an effective damping in the time dependent signal, shown in Fig. (S4).

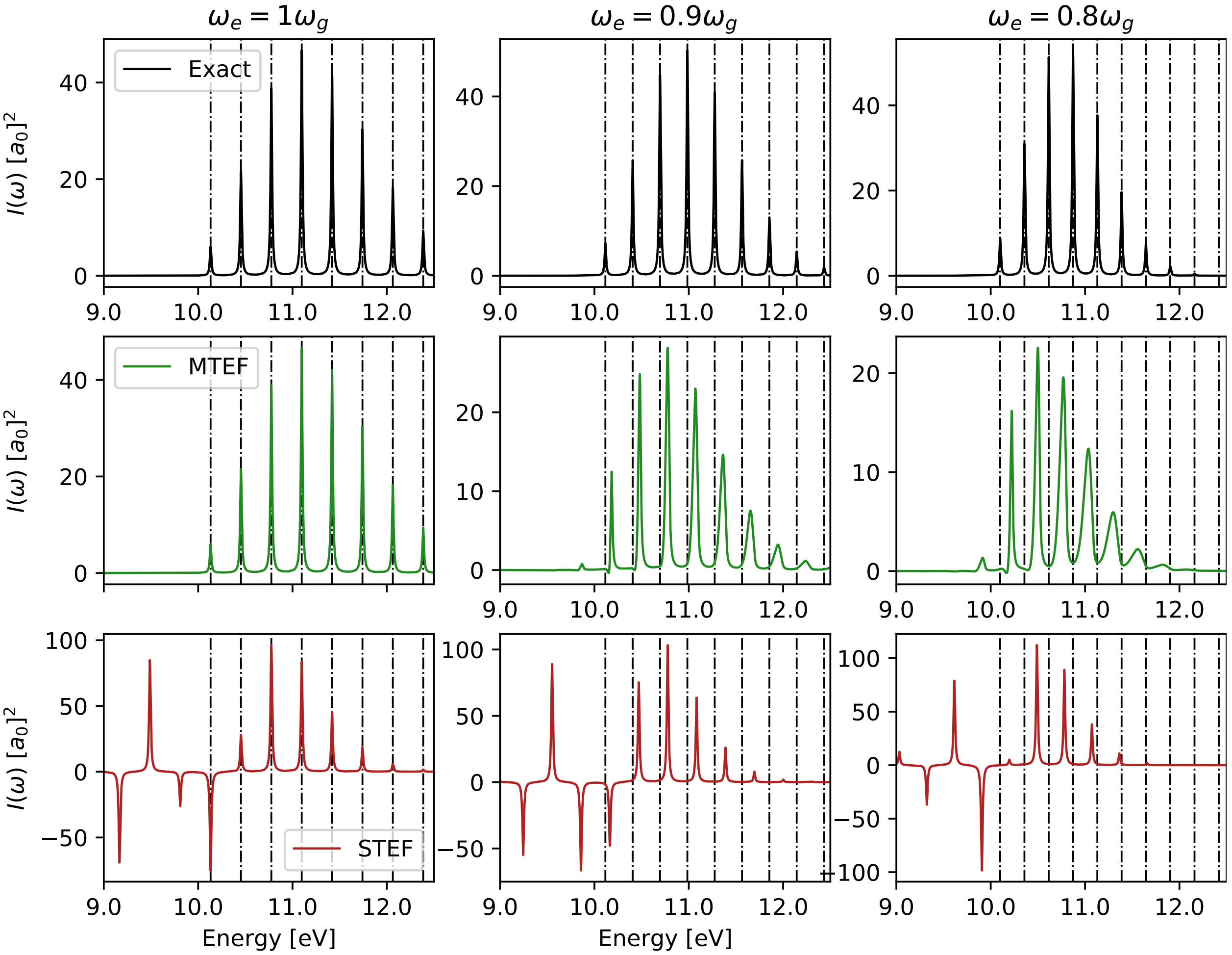

Figure S3: Spectra for the DHO model with several excited and ground state surface frequencies in each column. Each row compares exact, MTEF-BO and STEF-BO results respectively, with the Exact peak placement for each column overlaid across each as vertical dashed lines.

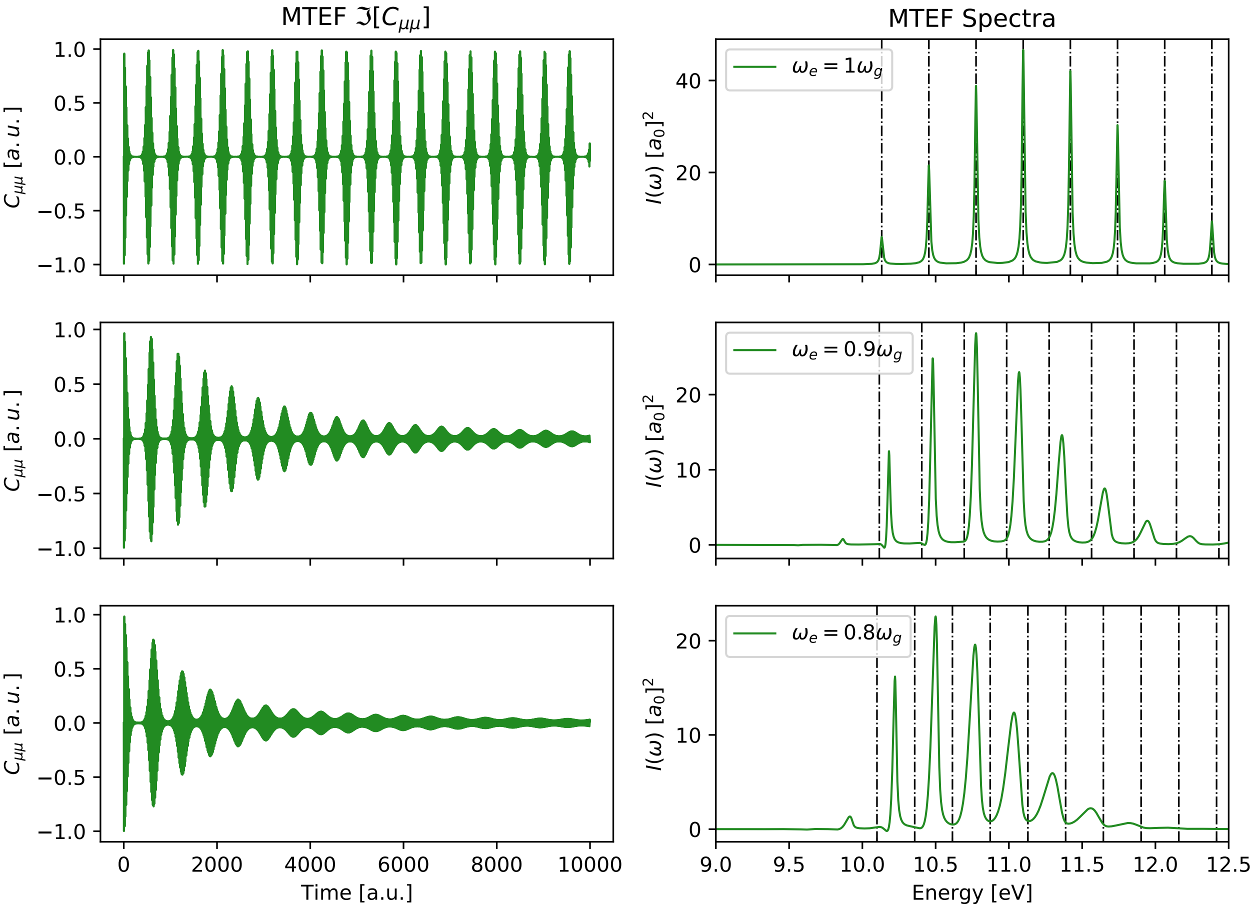

Figure S4: DHO MTEF time dependent dipole-dipole correlation signal in the left column and the resulting spectra in the right column, with the relative surface curvature denoted in the right column legend, and exact spectral peaks overlaid as vertical black dashed lines. For clarity, the time dependent signal is curtailed at $1 \cdot 10^4$a.u..

MTEF-Kick Comparison to the Nuclear Ensemble Approach

In the Nuclear Ensemble Approach (NEA)[7] the absorption spectra is written as

$$\begin{aligned}\sigma(\omega) &= \frac{4\pi^2}{c\omega}\sum_n \int dR|\chi_{00}(R)|^2\Delta\omega_{0n}^2(R)|\langle\phi_0|\mu_e(r,R)|\phi_n\rangle_r|^2 L(\omega-\omega_{0n}(R),\delta_n) \\ &= \frac{4\pi^2}{c\omega}\sum_n \frac{1}{N_t}\sum_{l=1}^{N_t}\Delta\omega_{0n}^2(R_l)|\langle\phi_0|\mu_e(r,R_l)|\phi_n\rangle_r|^2 L(\omega-\omega_{0n}(R_l),\delta_n) \\ L(x-x_i,\delta) &= \frac{1}{\pi}\frac{\delta}{(x-x_i)^2+(\delta)^2}\end{aligned} \tag{33}$$

Where $\Delta\omega_{0n}^2(R)$ is the vertical excitation energy between the ground and excited electronic states, μ_e is the electronic dipole operator, L is a Lorentzian broadening function dependent on a width parameter δ, and in the second line we have taken a Monte Carlo sampling integral of the first line, selecting R_l from $|\chi_{00}(R)|^2$. This Monte Carlo integral is precisely equivalent to the MTEF procedure sampling from the Wigner transform of the nuclear ground state, however unlike the MTEF-kick approach doesn't include any nuclear dynamics effects modulating the electronic properties. The NEA results for $N_t = 1.3\times10^5$ in the model H_2 system discussed in the main text are compared to the exact vibronic spectra and the MTEF-kick spectra in figure S5, showing directly that while sampling over initial equilibrium configuration naturally leads to a broadening of the resulting spectrum, the *dynamics* of the nuclear subsystem coupled to the electronic subsystem are responsible for the vibronic peak structure of the MTEF-kick results. A width parameter of $\delta = -\frac{\log(10^{-3})}{T_f\pi}$ was used for $T_f = 10,000$[a.u.], creating a width commensurate with the dynamics results which have a similar width due to the the exponential damping mask used in the Fourier transform of the dipole signal.

The NEA method was also applied to the benzene molecule, with the dipole oscillator strengths and transition energies calculated via the Casida equation with 400 unoccupied

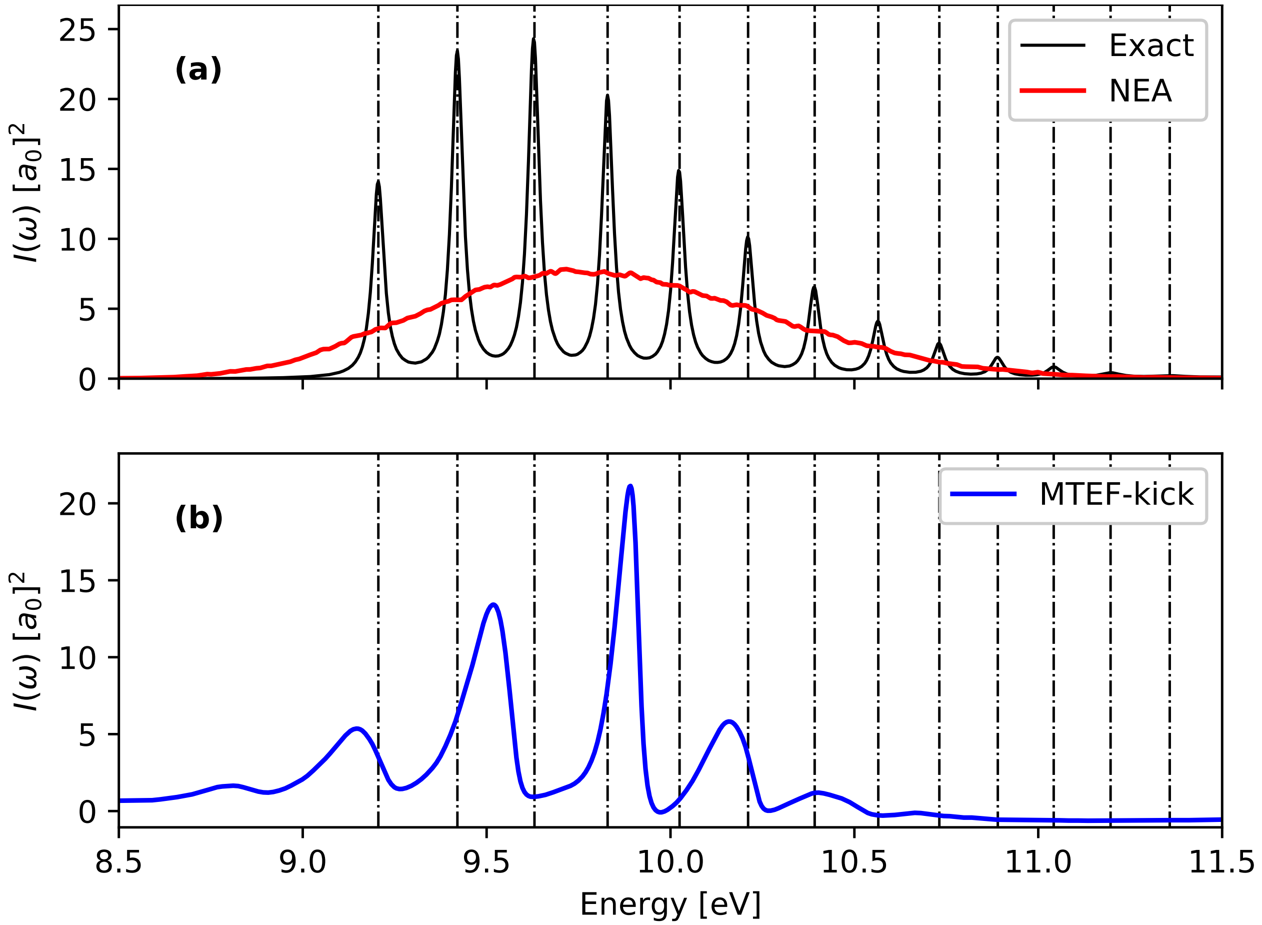

Figure S5: Exact vibronic spectra overlaid with the results of the NEA calculation in (a), with the MTEF-kick results from the main text recreated in (b).

orbitals for the same set of initial nuclear geometries used for the MTEF-TDDFT-kick spectra and the same simulation box parameters reported in the main text. The results are shown in figure S6. We see that there is good qualitative agreement between the two methods in this system, due to the density of electronic and vibrational states in the energy range. For completeness we also recreate the NEA results of Crespo-Otero and Barbatti[7] calculated with a more sophisticated xc functional, and find quite good agreement, particularly in the 5.5eV to 6eV energy range.

While these two methods are qualitatively quite similar for the linear optical absorption spectra, as evidenced from the NEA H_2 calculation generally speaking the MTEF dynamics do indeed add non-trivial information. Furthermore for non-linear and time dependent spectra and phenomena, static ion ensemble approaches like NEA are either inapplicable or less suitable than our proposed framework.

More Detail on the ICWF Method

The conditional wave function (CWF) approach can be developed starting from the full molecular wave function for electrons and nuclei, $\Psi(\mathbf{r}, \mathbf{R}, t)$, which can be formally decomposed in terms of the CWFs of each subsystem:

$$\psi_e^\alpha(\mathbf{r}, t) \;:=\; \int d\mathbf{R}\delta(\mathbf{R}^\alpha(t) - \mathbf{R})\Psi(\mathbf{r}, \mathbf{R}, t), \tag{34}$$

$$\psi_n^\alpha(\mathbf{R}, t) \;:=\; \int d\mathbf{r}\delta(\mathbf{r}^\alpha(t) - \mathbf{r})\Psi(\mathbf{r}, \mathbf{R}, t). \tag{35}$$

From these definitions one can show that the CWFs, $\psi_e^\alpha(t)$ and $\psi_n^\alpha(t)$, obey non-Hermitian equations of motion involving complex potentials which are functionals of the full wave function and cause the time-evolution of the individual CWFs to be non-unitary.[8] The recently developed Interacting-CWF (ICWF) method[9] avoids the direct calculation of these

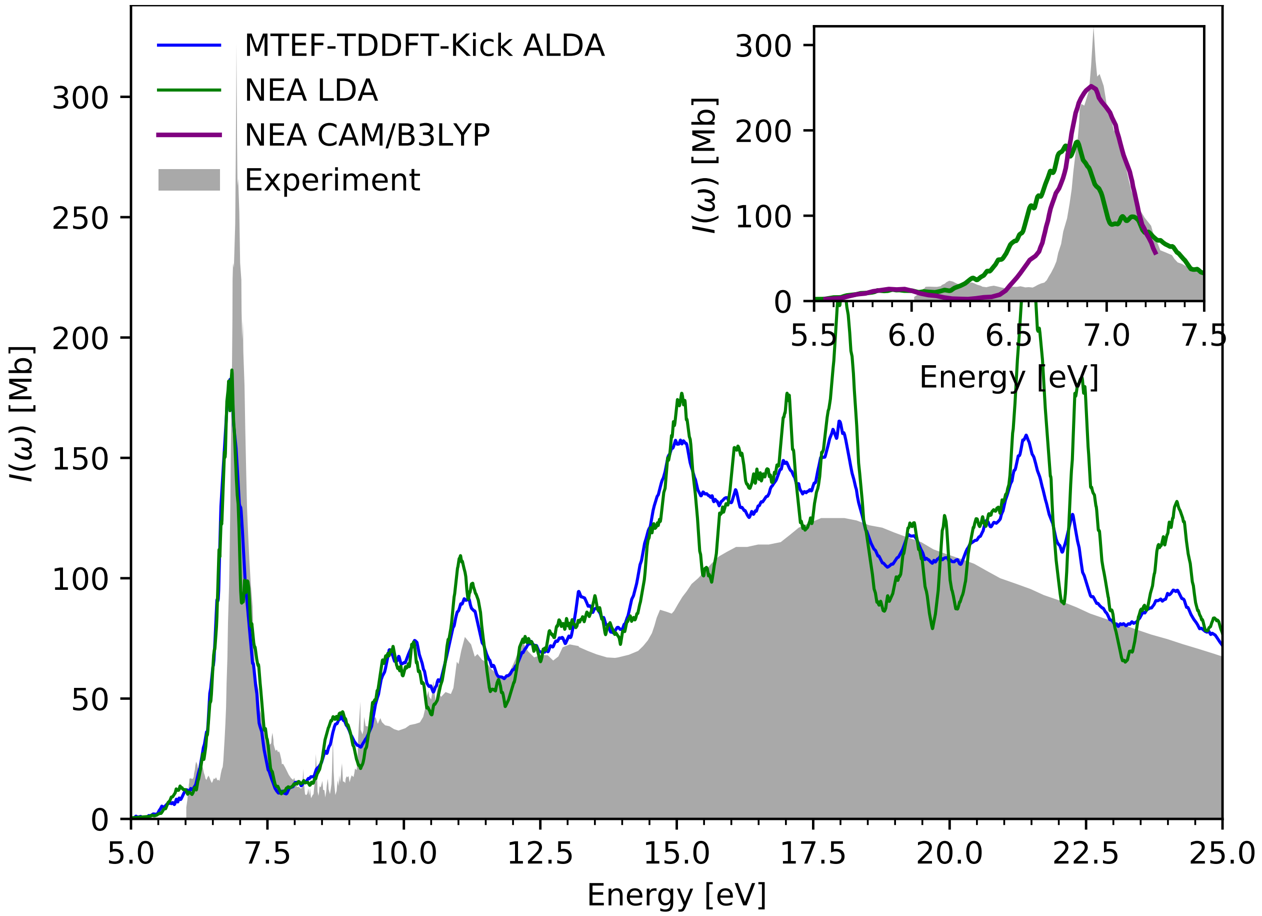

Figure S6: Benzene MTEF-TDDFT-kick spectrum calculated with the adiabatic LDA functional from the main text alongside the NEA spectrum calculated with the LDA functional and a CAM/B3LYP functional recreated from Crespo-Otero and Barbatti[7] compared to experiment.

nonlocal complex potentials by positing the following multiconfigurational CWF basis ansatz for the full many-body wave function:

$$\Psi(r, \mathbf{R}, t) = \sum_{\alpha=1}^{N_c} C_\alpha(t) \psi_e^\alpha(\mathbf{r}, t) \psi_n^\alpha(\mathbf{R}, t). \tag{36}$$

The basis functions in this sum are chosen to be single particle CWFs that satisfy the mean-field, or Hermitian, limit of the CWF equations in which the complex potentials trivially vanish. The upper limit of the sum, N_c, refers to the total number of configurations, which can be stochastically sampled. Including interactions between the trajectories in the ensemble through the coefficients $\mathbf{C}(t) = \{C_1(t), ..., C_{N_c}(t)\}$ corrects the Hermitian-CWF evolution. The time evolution of these coefficients is obtained by inserting eq. (36) directly into the TDSE.

As described in the text, for the kick spectra adapted ICWF algorithm, the CWFs are instead selected as eigenstates of the Hermitian propagators, and used as a static basis. The imaginary and real time equations of motion for the expansion coefficient $\vec{C}$ are then solved using the respective variational principles,[10–13] allowing for a completely closed-loop algorithm for wave function preparation and propagation.

To generate the kick spectra, after preparing the ground state $\vec{C}(0)$, the relevant degree of freedom of the kick operator $\exp(-i\kappa\hat{\mu})$ is applied to each CWF, the Hamiltonian and inverse overlap matrices are reconstructed, and $\vec{C}$ is propagated to the desired time. This procedure is equivalent to propagating in the interaction representation, with $\hat{V}_I(t) = \kappa\delta(t)\hat{\mu}$. Since these matrices are only constructed at time zero, this algorithm is extremely efficient, requiring only the propagation of a $N_c \times 1$ vector by a $N_c \times N_c$ matrix. For comparison, the 1D H2 MTEF-kick results reported here required the propagation of $34,000$ trajectories each consisting of $108^2 \times 1$ electronic wave functions. With a parallelized implementation and hardware allowing approximately 50traj/hr, this equates to roughly 680 compute hours. The ICWF $N_c = 4096$ results reported in the main body by contrast require 17 compute

hours on the same hardware.

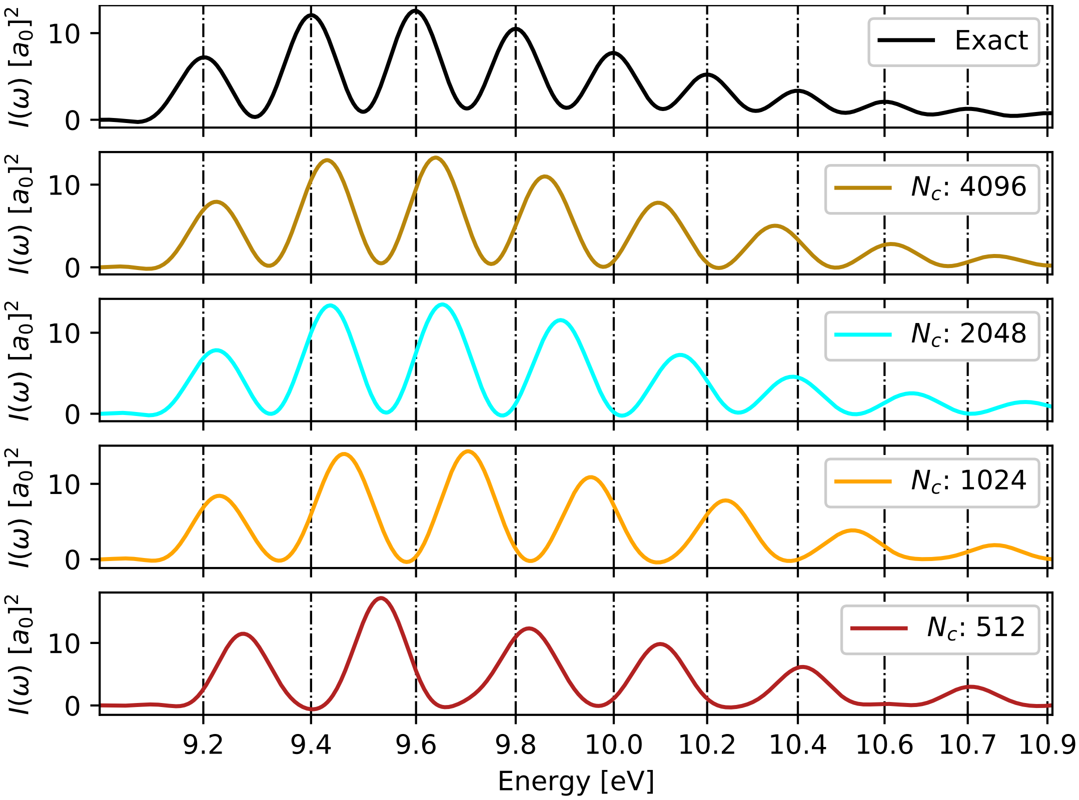

Figure S7: Convergence of the ICWF kick spectra results for increasing numbers of bases N_c. The two lowest lying peaks are mostly converged by $N_c = 1024$, but the higher energy spectra requires more variational degrees of freedom to capture.

With increasing non-redundant variational parameters, one is guaranteed to better capture the initial state and minimize the error of time dependent propagation.[12] As an example of the convergence properties of ICWF-kick, see Fig. S7. These spectra are the result of utilising only lowest energy hermitian propagator eigenstates and propagating for $T_f = 1500$ a.u. with a mask function[14] $W(x) = 1 - 3x^2 + 2x^3$, for $x = t/T_f$ applied to the time signal in the Fourier Transform. The more accurate $N_c = 4096$ results in the main body are initialised using mixes of the three lowest energy CWF eigenstates in roughly equal proportions.

Theoretical and practical developments are underway to implement the ICWF method in arbitrary ab-initio settings.

Complex Absorbing Potentials

Quadratic complex absorbing potentials[15] of the following form were used in all simulations of the one dimensional H_2 model:

$$\begin{aligned} W_e(r_i) &= -i\eta\left[(r_i - r_l)^2\Theta(r_l - r_i) + (r_i - r_r)^2\Theta(r_i - r_r)\right] \\ W_n(R) &= -i\eta(R - R_r)^2\Theta(R - R_0), \end{aligned} \tag{37}$$

where Θ is the Heaviside function, and η was set to $0.1\text{Ha}/a_0$ for both subsystems.

The electronic CAP cut offs, r_l and r_r, were placed $10a_0$ from the walls, while the nuclear CAP start was set at $R_0 = 5.6875a_0$.

References

(1) Fairlie, D. B. Moyal brackets, star products and the generalized Wigner function. *Chaos, solitons and fractals* **1999**, *10*, 365–371.

(2) Kapral, R. Mixed quantum-classical dynamics. *Journal of Chemical Physics* **1999**, *110*, 8919–8929.

(3) Case, W. B. Wigner functions and Weyl transforms for pedestrians. *American Journal of Physics* **2008**, *76*, 937–946.

(4) Goings, J. J.; Lingerfelt, D. B.; Li, X. Can Quantized Vibrational Effects Be Obtained from Ehrenfest Mixed Quantum-Classical Dynamics? *Journal of Physical Chemistry Letters* **2016**, *7*, 5193–5197.

(5) Tokmakoff, A. Time-Dependent Quantum Mechanics and Spectroscopy. *Lecture* **2014**,

(6) McKemmish, L. K.; McKenzie, R. H.; Hush, N. S.; Reimers, J. R. Quantum entanglement between electronic and vibrational degrees of freedom in molecules. *Journal of Chemical Physics* **2011**,

(7) Crespo-Otero, R.; Barbatti, M. Spectrum simulation and decomposition with nuclear ensemble: Formal derivation and application to benzene, furan and 2-phenylfuran. *Theoretical Chemistry Accounts* **2012**, *131*, 1–14.

(8) Albareda, G.; Appel, H.; Franco, I.; Abedi, A.; Rubio, A. Correlated electron-nuclear dynamics with conditional wave functions. *Physical Review Letters* **2014**,

(9) Albareda, G.; Kelly, A.; Rubio, A. Nonadiabatic quantum dynamics without potential energy surfaces. *Physical Review Materials* **2019**, *3*.

(10) Shi, T.; Demler, E.; Ignacio Cirac, J. Variational study of fermionic and bosonic systems with non-Gaussian states: Theory and applications. *Annals of Physics* **2018**, *390*, 245–302.

(11) Broeckhove, J.; Lathouwers, L.; Kesteloot, E.; Van Leuven, P. On the equivalence of time-dependent variational principles. *Chemical Physics Letters* **1988**, *149*, 547–550.

(12) Lubich, C. On variational approximations in quantum molecular dynamics. *Mathematics of Computation* **2004**, *74*, 765–780.

(13) Ohta, K. Time-dependent variational principle with constraints for parametrized wave functions. *Physical Review A - Atomic, Molecular, and Optical Physics* **2004**, *70*.

(14) Yabana, K.; Nakatsukasa, T.; Iwata, J. I.; Bertsch, G. F. Real-time, real-space implementation of the linear response time-dependent density-functional theory. *Physica Status Solidi (B) Basic Research* **2006**, *243*, 1121–1138.

(15) Muga, J. G.; Palao, J. P.; Navarro, B.; Egusquiza, I. L. Complex absorbing potentials. 2004.
